# Budget impact analysis of two treatment approaches for hepatitis C in Malaysia through the use of voluntary and compulsory licensing options

**DOI:** 10.3389/fpubh.2023.1114560

**Published:** 2023-02-22

**Authors:** Amirah Azzeri, Maznah Dahlui, Rosmawati Mohamed, Scott Alexander McDonald, Hafiz Jaafar, Fatiha Hana Shabaruddin

**Affiliations:** ^1^Public Health Unit, Department of Primary Health Care, Faculty of Medicine and Health Sciences, Universiti Sains Islam Malaysia, Nilai, Negeri Sembilan, Malaysia; ^2^Department of Research, Development and Innovation, University Malaya Medical Centre, Kuala Lumpur, Malaysia; ^3^Centre of Population Health, Department of Social and Preventive Medicine, Faculty of Medicine, University Malaya, Kuala Lumpur, Malaysia; ^4^Faculty of Public Health, Universitas Airlangga, Surabaya, Indonesia; ^5^Department of Medicine, Faculty of Medicine, University Malaya, Kuala Lumpur, Malaysia; ^6^Centre for Infectious Disease Control, National Institute for Public Health and the Environment, Bilthoven, Netherlands; ^7^School of Health and Life Sciences, Glasgow Caledonian University, Glasgow, United Kingdom; ^8^Faculty of Pharmacy, University Malaya, Kuala Lumpur, Malaysia

**Keywords:** hepatitis C, direct acting antiviral, economic burden, budget impact analysis, Malaysia

## Abstract

**Introduction:**

A scaled-up treatment cascade with direct-acting antiviral (DAA) therapy is necessary to achieve global WHO targets for hepatitis C virus (HCV) elimination in Malaysia. Recently, limited access to sofosbuvir/daclatasvir (SOF/DAC) is available through compulsory licensing, with access to sofosbuvir/velpatasvir (SOF/VEL) expected through voluntary licensing due to recent agreements. SOF/VEL has superior clinical outcomes but has higher drug acquisition costs compared to SOF/DAC. A stratified treatment cascade might be the most cost-efficient approach for Malaysia whereby all HCV patients are treated with SOF/DAC except for patients with cirrhosis who are treated with SOF/VEL.

**Methods:**

This study aimed to conduct a 5-year budget impact analysis of the proposed stratified treatment cascade for HCV treatment in Malaysia. A disease progression model that was developed based on model-predicted HCV epidemiology data was used for the analysis, where all HCV patients in scenario A were treated with SOF/DAC for all disease stages while in scenario B, SOF/DAC was used only for non-cirrhotic patients and SOF/VEL was used for the cirrhotic patients. Healthcare costs associated with DAA therapy and disease stage monitoring were included to estimate the downstream cost implications.

**Results:**

The stratified treatment cascade with 109 in Scenario B was found to be cost-saving compared to Scenario A. The cumulative savings for the stratified treatment cascade was USD 1.4 million over 5 years.

**Discussion:**

A stratified treatment cascade with SOF/VEL was expected to be cost-saving and can result in a budget impact reduction in overall healthcare expenditure in Malaysia.

## 1. Introduction

Chronic hepatitis C viral (HCV) infection and its complications carries huge costs to individual patients, affected households and healthcare providers (1, 2). The cost of HCV drugs constitutes the largest proportion ranging from 85 to 90% of the total direct medical costs and the cost has escalated further with the use of new antiviral drugs (3, 4).

Direct Acting Antiviral (DAA) drugs are extremely expensive and are unaffordable for many countries, especially those that do not have access to generic formulations and fall outside of license agreements (5). It is estimated that, if DAA drugs were used for all hepatitis C infected patients in the national healthcare system, some countries would spend half of their annual total pharmaceutical expenditure on DAA alone (6), which is clearly unaffordable. Making effective and affordable hepatitis C drugs available within a health care system is a crucial move to improve the health outcome of infected patients.

Globally, DAA drugs are now widely used for the treatment of HCV infection. The predominant HCV genotypes in Malaysia are genotype 3 followed by genotype 1, accounting for 99% of all HCV cases (7, 8). This implies that any DAA regimen with proven efficacy for these two genotypes could be adopted within a simple management protocol. Standard use of such DAA regimens for all non-cirrhotic and cirrhotic patients would remove the need for genotyping and thus will further expedite initiation of treatment for all HCV-infected patients. Hypothetically, if DAA is available as standard HCV treatment for this study population (as compared to the previously use of INF), the proportion of patients who would be eligible for HCV treatment will rise due to the wider eligibility for DAA treatment for patients, including those with decompensated cirrhosis, assuming no other restricting factors for treatment initiation, such as refusal and loss of follow-up.

Improved access to DAA drugs (sofosbuvir/daclatasvir and sofosbuvir/velpatasvir) through both government-use compulsory licensing and voluntary licensing would significantly enhance the hepatitis C treatment program in Malaysia. Both regimens comprise two out of three pangenotypic DAA medicines that are strongly recommended by the recent WHO guidelines due to their high treatment efficacy across all HCV genotypes, including among patients with cirrhosis or HIV coinfection.

Malaysia has taken a unique path to accessing generic DAA drugs for HCV patients. Initial negotiations for the country to be included in the voluntary license agreement with the sole DAA innovator company were unsuccessful because Malaysia was considered to be an upper-middle-income country (9). This led to the Malaysian government's decision to issue a government-use license to produce generic sofosbuvir, one of the main drugs in DAA combination regimens used to treat HCV. Through the use of compulsory licensing, the government of Malaysia aimed to treat patients in stages in 5 years, using the sofosbuvir/daclatasvir combination therapy. Current treatment program was planned to use existing resources in healthcare facilities without expansion of the national screening and treatment program (10). This plan, however, will limit the number of treatment recipients to fewer than 20,000 over 5 years, which may be feasible to achieve but is claimed to be insufficient to accomplish the global target of hepatitis C elimination as a public health threat by 2030 (11). Following the granting of the government-use license, Malaysia was included in the voluntary license agreement list, which led to additional DAA options, including sofosbuvir/velpatasvir combination therapy, thereby providing an opportunity to extend national HCV treatment access beyond government hospitals to include university and private hospitals (12). A national treatment program using both combination drugs might be the most cost-efficient option to expand treatment access and optimize healthcare spending on DAA drugs while minimizing downstream clinical consequences in Malaysia (9).

It has been estimated that the acquisition cost for the generic sofosbuvir/daclatasvir obtained through the government compulsory license is lower than that of the generic sofosbuvir/velpatasvir obtained through voluntary license (10, 13). However, the need for longer treatment duration results in higher overall treatment cost with sofosbuvir/daclatasvir compared to sofosbuvir/velpatasvir particularly in cirrhotic patients. In view of the comparable outcomes of both combination drugs and the cost differences, a stratified treatment cascade was proposed of which sofosbuvir/daclatasvir for all HCV non-cirrhotic patients and sofosbuvir/velpatasvir for cirrhotic patients (13).

To date, the economic implications of the mix of interventions (sofosbuvir/daclatasvir and sofosbuvir/velpatasvir) through the proposed stratified treatment cascade in national HCV treatment program is unknown. Therefore, it is essential for Malaysia to conduct its own context-specific economic assessment from the Government's perspective to estimate the fiscal impact of HCV treatment on the national healthcare budget. Therefore, a budget impact analysis was conducted to describe the economic consequences of using available DAA drugs comparing current treatment program and the proposed stratified treatment approach for HCV treatment in the country. The findings from this study can inform the Ministry of Health Malaysia (MoH)'s decision-making in relation to resource reallocation and policy development for hepatitis C disease. They can also contribute to the development of local clinical guidelines and a local treatment strategy for hepatitis C patients, which in turn can lead to better control of the disease. Information on the estimated impact on the health budget can facilitate future planning and funding for HCV disease in Malaysia.

## 2. Materials and methods

A Budget Impact Analysis (BIA) estimates the changes in healthcare expenditure for a budget holder following a new or additional healthcare intervention. It describes the short-term healthcare expenditure to fund the intervention (14). A BIA is useful for financial sustainability and healthcare budget planning (15). Due to the growing interest in BIA, many international guidelines have been developed since 2000 to provide a standard approach for the analysis (16).

This BIA reflected the change in Ministry of Health expenditure for hepatitis C management with DAA therapy over 5 years, from 2018 to 2022. The BIA conducted in this study reflected the current public healthcare facilities in Malaysia based on the availability and capability of healthcare services for hepatitis C disease through existing programs and resources, without expansion of the screening and treatment program. A 5-year assessment was conducted as recommended by recent international guidelines for budget impact analysis and the assessment started in the year 2018 following the agreement of the government-use compulsory license for hepatitis C treatment program in Malaysia in late 2017. The assessment was conducted in Microsoft Excel as recommended by recent guideline (16).

In the analysis, two scenarios were explored. The only difference between the two scenarios was the use of different DAA-combination drugs for cirrhotic patients. Otherwise the non-cirrhotic patients receive the same DAA drug in both scenarios. The number and characteristics of patients were identical in both scenarios and did not include new HCV patients in public healthcare facilities over the 5-year period. Although the availability of hepatitis C treatment through voluntary licensing might allow for more patients to be in care and to be treated, especially at private healthcare settings, this was beyond the scope of this analysis. The assessment was informed and populated by several parameters as described below.

The input parameters included in the BIA framework were: (i) number and characteristics of HCV in care and number of patients receiving DAA treatment; (ii) the different DAA treatment scenarios; and (iii) healthcare costs of the different strategies, which included treatment cost and disease stage-related cost.

### 2.1. Number and characteristics of hepatitis C patients in care and number of patients receiving DAA treatment

Both number and characteristics of hepatitis C patients in care and number of patients receiving DAA treatment were derived from series of modeling studies (11, 17, 18). The numbers were estimated for all disease stages relevant to HCV, which include non-cirrhotic chronic infection (NCCI), compensated cirrhosis (CC), decompensated cirrhosis (DC), and hepatocellularcarcinoma (HCC). Initially, previous works that estimated the prevalence and disease burden of hepatitis C infection in Malaysia was conducted and the initial modeling work estimated the national prevalence of hepatitis C disease based on available related epidemiological data, such as gender distribution, age group, risk factors, and HCV-HIV co-infection (17). Following the initial modeling work, second modeling work projected the burden of HCV-related complications in Malaysia up to the year 2039 by incorporating additional information to the first model such as annual transition probabilities of HCV disease progression stages, mortality rates, antiviral treatment uptake and sustained virological response rates (18).

Following findings from both studies (17, 18), the third modeling work was conducted to estimate the annual number and characteristics of patients in clinical care and the number of patient initiated DAA treatment in Malaysia (11). The recent modeling work projected the number of patients within care, annual numbers of patients initiated on antiviral treatment, and distribution of treatments for eligible stages of liver disease, for four possible treatment scenarios: (a) no antiviral treatment offered; (b) previous antiviral treatment with PEGINF/RBV; (c) scale-up in DAA treatment uptake deemed achievable, but not meeting the global treatment uptake target set by World Health Organization (WHO); and (d) scale-up in DAA treatment meeting the global treatment target set by WHO (11). For the purpose of this BIA analysis, scenario (c) was selected in line with the MoH's goals for hepatitis C treatment in Malaysia (10).

Distribution of patients according to disease stages reported earlier (19) were used together with information from experts to inform and populate the analysis. In the modeling work, it was assumed that only symptomatic patients were in care, and estimated that 15% of patients in NCCI stage of liver disease, 60% of patients in CC stage of liver disease, and 100% of patients in DC and HCC stage of liver disease were symptomatic. Of the symptomatic patients, it was postulated that 10, 70, and 100% of symptomatic patients in NCCI stage of liver disease, CC stage of liver disease and DC and HCC stage of liver diseases, respectively, were in care and, therefore, incurred costs in public healthcare services in Malaysia. Treatment distributions for each disease stage of liver disease were gathered from expert opinion and were estimated at 70% for NCCI, 26% for CC, and 4% for DC stages of liver diseases. Patients in cirrhotic stages of liver diseases who were successfully cured from the infection and patients in the HCC stage of liver disease remained in care, as these patients still required clinical monitoring for liver conditions even when they were cured, based on standard clinical pathways.

The number and characteristics of hepatitis C patients in care and the number of patients receiving DAA treatment in Malaysia were projected from the year 2018 to 2022. A 5-year economic assessment was conducted as recommended by recent international guidelines for budget impact analysis (16). The assessment started in the year 2018 following the agreement of the government-use compulsory license for hepatitis C treatment program in Malaysia in late 2017. Through this recent agreement, DAA therapy was distributed at 22 public hospitals. The annual number of patients in clinical care and the number of patient-initiated DAA treatments between 2018 and 2022 were presented in Table 1.

**Table 1:** Annual number of patients within care and number of patient initiated DAA treatment in Malaysia for 2018 until 2022.

	**Number of patients within care**					**Number of patient initiated treatment**			
**Disease stage of liver disease**	**Non-cirrhotic chronic infection**	**Compensated cirrhosis**	**Decompensated cirrhosis**	**Hepatocellular carcinoma**	**Total**	**Non-cirrhotic chronic infection**	**Compensated cirrhosis**	**Decompensated cirrhosis**	**Total**
**Year**									
2018	5,398	10,451	6,039	640	22,528	1,400	520	80	2,000
2019	5,427	10,664	6,137	649	22,877	1,750	650	100	2,500
2020	5,442	10,874	6,210	655	23,181	2,450	910	140	3,500
2021	5,445	11,083	6,250	658	23,436	3,150	1,170	180	4,500
2022	5,434	11,289	6,252	657	23,632	3,850	1,430	220	5,500

### 2.2. Examination of different interventions

Two different treatment scenarios were compared. Scenario A reflected the current national treatment scenario of sofosbuvir/daclatasvir for eligible hepatitis C patients in pre-carcinoma disease stages (including NCCI, CC and DC), while Scenario B described a stratified treatment cascade where sofosbuvir/daclatasvir was used for all patients, except those with CC and DC, who were expected to receive sofosbuvir/velpatasvir. The clinical pathway for DAA treatment for different disease stages of liver diseases was used to estimate the resource used and hence the medical costs (20). For scenario B, the use of sofosbuvir/velpatasvir for patients in the NCCI stage of liver disease was similar to that in scenario A, while the sofosbuvir/velpatasvir regimen for CC and DC stages of liver diseases was for 12 weeks, with the addition of RBV for decompensated cirrhosis patients as described in the recent WHO guideline (21). Otherwise, the frequency and type of investigation during and after the course of therapy were similar for both combination DAA drugs.

For both scenarios the clinical pathway for the treatment was obtained from clinical practice guideline (20), expert opinion and recent WHO guideline (21). In the BIA model, all patients were assumed to receive one course of DAA therapy only to reflect the current clinical scenario in Malaysia.

### 2.3. Healthcare costs of the different treatment scenarios

The related healthcare costs included in the BIA were the treatment cost (1st year treatment cost) with DAA drugs and disease stage management-related cost. The healthcare cost applied in this BIA, were primarily calculated by using combination of macrocosting and activity-based costing methods. The costing study was conducted at Selayang Hospital, the national referral center for liver disease in Malaysia. The details of the costing study were described elsewhere (11). All costs were inflation-adjusted to reflect costs in 2021 using the consumer price index factor (22) and were presented in the local currency, Malaysian Ringgit (RM); at the time of reporting, US$ 1 = RM 4.14. All the costs used in this analysis are presented in Table 2.

**Table 2:** Annual treatment cost (1st year treatment cost) with DAA drugs and disease stage management-related cost (USD, 2021).

**First year treatment cost with DAA drugs (sofosbuvir/daclatasvir)**						
	**Outpatient visits**			**Laboratory investigations**	**Sofosbuvir/daclatasvir**	**Total**
Non-cirrhotic decompensated cirrhosis	199.4			153.58	299.94	652.92
Compensated cirrhosis	199.4			153.58	599.88	952.86
Decompensated cirrhosis	465.27			185.21	2,673.07	3,323.55
**Annual recurring cost of disease stage monitoring**						
	**Outpatient**	**Daycare**	**Hospital**	**Laboratory**	**Regular medication other**	**Total**
	**visits**	**visits**	**admissions**	**investigations**	**than DAA drugs**	
Non-cirrhotic decompensated cirrhosis	132.93			82.00		214.93
Compensated cirrhosis	132.93	107.17		27.55		267.65
Decompensated cirrhosis	265.87	815.94	4,724.93	48.85	140.09	5,995.68
Hepatocelularcarcinoma (HCC)						6,415.68^*^
**First year treatment cost with DAA drugs (sofosbuvir/velpatasvir)**						
	**Outpatient**			**Laboratory**	**Sofosbuvir/velpatasvir**	**Total**
	**visit**			**investigation**		
Non-cirrhotic decompensated cirrhosis	199.4			153.58	479.9	832.88
Compensated cirrhosis	199.4			153.58	479.9	832.88
Decompensated cirrhosis	465.27			185.21	1,516.5	2,166.98

^*^The weighted average annual cost of HCC management across different treatment modalities [include HCC treated with radiofrequency ablation (RFA), HCC treated with transarterial chemoembolization (TACE) with or without DC beads and HCC treated with liver resection] with cirrhosis.

This cost estimate was based on one course of DAA therapy only. The efficacy of DAA therapy in achieving cure rates of nearly 100% suggests that treatment failure with DAA is very rare, and failures are commonly associated with poor treatment adherence and drug-drug interactions (21). In addition, all costs related to adverse events were excluded. Even though the clinical wellbeing of particular patients intra-treatment may necessitate frequent and more extensive utilization than is suggested in the clinical pathways, particularly with RBV, treatment with DAA therapy generally has very low rates of adverse events (21).

## 3. Results

The annual cost of treating hepatitis C patients in Malaysia with Scenario A, the current national treatment scenario of sofosbuvir/daclatasvir for eligible hepatitis C patients in pre-carcinoma disease stages, was estimated to be ~USD 46 million in the 1st year, USD 47 million in the 2nd year, USD 49 million in the 3rd year, USD 50 million in the 4th year, and USD 51 million in the 5th year.

For Scenario B, the proposed stratified treatment cascade where sofosbuvir/daclatasvir was used for all patients, except those with CC and DC, who were expected to receive sofosbuvir/velpatasvir, the annual cost of treating hepatitis C patients in Malaysia was estimated to be USD 46 million in the 1st year, USD 47 million in the 2nd year, USD 49 million in the 3rd year, USD 50 million in the 4th year, and USD 50 million in the 5th year. In general the assessment found that Scenario B incurred less cost throughout the 5 years of the analysis compared to scenario A. The cost differences for hepatitis C treatment in scenarios A and B in the 5 years of the analysis were USD 157 thousand, USD 196 thousand, USD 275 thousand, USD 354 thousand, and USD 433 thousand, respectively. The cumulative savings over the 5 years were estimated to be USD 1.4 million. The results of the budget impact analysis between scenario A and scenario B for hepatitis C treatment in Malaysia are presented in Table 3. In both scenarios, the share of disease stage management decreased over the 5 years from 94 to 86% and the share for treatment cost increased from 6 to 13% from 2018 to 2022.

**Table 3:** Budget impact analysis over 5 years for treating chronic hepatitis C patients with sofosbuvir/daclatasvir (scenario A) and stratified treatment cascade which sofosbuvir/daclatasvir in pre-cirrhotic stage and sofosbuvir/velpatasvir in cirrhotic stage (scenario B; USD, 2021).

		**Cost of hepatitis C treatment**					
		**Year 2018**	**Year 2019**	**Year 2020**	**Year 2021**	**Year 2022**	**Total**
**Scenario**							
Sofosbuvir/daclatasvir for patients in all eligible disease stages	Annual healthcare cost	46,737,718.80	47,884,552.33	49,281,534.27	50,454,778.94	51,366,284.05	245,724,868.39
Proposed stratified treatment cascade where sofosbuvir/daclatasvir for NCCI patients and sofosbuvir/velpatasvir for CC and DC patients	Annual healthcare cost	46,580,139.00	47,687,577.58	49,005,769.63	50,100,224.40	50,932,939.59	244,306,650.20
Cost difference		157,579.80	196,974.75	275,764.64	354,554.54	433,344.46	1,418,218.19

## 4. Discussion

The budget impact analysis of two different hepatitis C treatment scenarios estimated that a stratified treatment cascade with sofosbuvir/daclatasvir and sofosbuvir/velpatasvir was more cost-efficient. The total cost of USD 245 billion was found for the treatment scenario with sofosbuvir/daclatasvir, compared to USD 244 billion for the stratified treatment cascade with sofosbuvir/daclatasvir and sofosbuvir/velpatasvir over 5 years from 2018 to 2022. The cumulative savings for the stratified treatment cascade was USD 1.4 million over 5 the years, which mainly due to the shorter treatment duration with sofosbuvir/velpatasvir for cirrhotic patients.

The current HCV treatment program in Malaysia, based on existing resources without the expansion of the screening and treatment program, will limit the number of treatment recipients to fewer than 20,000 over the 5 years, which may be feasible to achieve but is claimed to be insufficient to achieve the global target of hepatitis C elimination as a public health threat by 2030 due to the gradual scale-up in annual treatment uptake (11).

The voluntary licensing agreement is expected to make an important contribution to increasing nationwide access to HCV treatment in hepatitis C-infected individuals in Malaysia (12). At the moment, the cost saving of the stratified treatment approach is relatively small at the current price of sofosbuvir/velpatasvir. It is expected that the voluntary DAA licensing agreement in Malaysia will lead to further reduction of DAA acquisition cost through competitive market pricing by several manufacturers of generic DAA drugs, leading to further savings for the Ministry of Health. It is anticipated that the acquisition cost of DAA will be reduced in Malaysia in subsequent years. A previous projection indicated that the total healthcare cost of HCV management was most sensitive to the pricing of DAA and reduction of the cost of DAA by one-third and two-thirds of the current price would lower the average cost of treatment by 9 and 18%, respectively (11). In this study, an extended analysis was conducted by reducing the costs of sofosbuvir/velpatasvir given the possible reduction in the market price of the drug through the competitive market. When the cost of the sofosbuvir/velpatasvir drug is reduced by 10, 20, and 50% from the current price of USD480 per treatment, the cumulative savings over the 5 years between scenarios A and B were estimated to be USD 1.6, 2, and 3 million, respectively. This study found that DAA drug was the key cost driver across all disease stages, ranging from 46 to 80% of the cost, which is consistent with findings from previous studies (4, 21, 23). Similarly, in the era of interferon-based treatment, HCV drug cost was also found to be the main driver of high treatment cost (4, 22). The higher cost of HCV treatment with interferon-based drugs was related to the need for multiple courses of therapy and extended treatment duration (4) due to the low efficacy and higher failure rates of interferon. In contrast, the cost of DAA is exorbitant because of the monopoly held by pharmaceutical companies that ware protecting their intellectual property rights on patent-protected medications and the excessive profits generated by drug innovation (6), even though the actual cost of production and manufacturing the medication has been found to be low (24, 25).

Hepatitis C treatment scenario in Malaysia is unique. Not only was Malaysia the first country in the world to invoke compulsory licensing for the generic version of DAA, the main developer of DAA has also decided to include Malaysia in the voluntary licensing scheme. At the same time, Malaysia will also have access to another type of generic DAA drug, which is Ravidasvir. Recently, it was announced that one of Malaysian pharmaceutical companies, Pharmaniaga, agreed to support the registration of Ravidasvir for HCV treatment and MoH has decided to expedite the process of the registration in the national formulary (26). Soon, the new HCV treatment, Ravidasvir will be distributed to eligible patients and is to be used with a generic Sofosbuvir. The developer of Ravidasvir agreed to set the commercial price at $294 or less per treatment course, which is lower than the cost of generic DAA drugs through compulsory licensing and voluntary licensing scheme (27). At the moment, Ravidasvir is not in WHO essential medicine list (28). Nevertheless, it has potential pangenotypic criteria and shown to have excellent efficacy and safety across HCV disease stages (29). When the Sofosbuvir/Ravidasvir combination drugs are publicly available in the healthcare facilities in Malaysia, another budget impact analysis would help to assess the financial implications of HCV treatment which can inform policy planning and budgeting for the development of a sustainable hepatitis C program in the context of limited economic resources to optimize benefits for the healthcare system and HCV patients in Malaysia.

The 3-fold cost-reduction strategies for DAA pricing in Malaysia would help to reduce costs for DAA drugs and simultaneously help to widen the access to HCV treatment. It was estimated that, a scale-up of HCV treatment is essential to maximize the benefits of access to affordable DAA therapy in Malaysia, with a steep scale-up in the annual number of treatments initiated being essential for that country to achieve the elimination targets. Annual treatment initiation needs to increase from 5,000 patients in 2018 to 15,000 patients by 2022, and then rapidly scale up further to reach 30,000 by 2025. When most eligible patients have been treated, it is estimated that treatment initiation can reduce to 25,000 per year by 2029 and 2030 (11). Such a scale-up in treatment uptake is only feasible when conducted concurrently with a large-scale national screening program to prevent saturation of the known patient pool and to bring sufficient undiagnosed chronically infected patients into the treatment pathway (13).

While affordable acquisition costs of HCV treatment for all hepatitis C infected populations are necessary, there is also a need to improve the continuum of hepatitis C care (through screening, early diagnosis, timely linkage to and retention in care) as part of the strategy to achieve the WHO global targets for HCV elimination (12). Generally, a wide-scale screening approach is necessary to achieve the target of a 90% diagnosis rate of hepatitis C by 2030. For Malaysia, with an estimated 380,000 HCV infected individuals, of whom more than 90% remain undiagnosed and untreated, minimizing HCV-related costs remains challenging even with the availability of affordable DAA drugs. Identifying more patients with active infection is necessary. The medical costs for HCV infection will keep escalating, especially among patients who remain untreated, as they are at the highest risk of progressing to advanced disease stages and liver-related death. As these individuals with active infection grow old and the disease becomes advanced, this cohort of the HCV-infected population will incur the highest costs of treatment. A projection shows that a steep rise in the annual DAA treatment uptake through a comprehensive screening program would result in a decline in annual costs from the year 2025 onwards, while a limited number of patients treated with DAAs would produce a continuous increase in healthcare costs from the base-case cost in 2018 until 2040 (11).

Implementing national HCV screening program in Malaysia will require substantial national investment. In order for Malaysia to achieve the targets set by the WHO, a step-wise approach to a national screening strategy was proposed which relied on targeting people who inject drugs PWID in the early years, with delayed initiation of widespread screening of the general population. It will require 6.9 million people to be screened between 2018 and 2030, with general population screening to commence in 2025, to yield sufficient numbers of treatment-initiated patients (30). The cumulative cost was projected at RM241 million [USD62 million] (price year 2018) from 2018 to 2030. It was estimated that 10% of the total HCV program cost in Malaysia is required for screening activities alone. As the prices of DAA drugs fall, the screening program will represent a huge proportion of the overall HCV program cost compared to the costs of drugs and disease stage monitoring (31). In order to ameliorate the financial repercussions of the program, it was recommended that the HCV screening strategies should be integrated with existing healthcare resources and services. Such programmatic synergies would yield financial and infrastructural efficiencies compared to developing a separate program for HCV (30).

However, this analysis was conducted before the COVID-19 pandemic hit the country. The COVID-19 pandemic that has occurred over the past 3 years has resulted in several challenges for Malaysia to provide services to hepatitis C patients as usual. During the spike in the cases of COVID-19, some services at public and private healthcare facilities had to be limited to make room for COVID-19 services. Visits to specialist clinics had to be restricted and elective treatments were postponed (32). Nevertheless, as the country is moving toward endemic with the vaccination rates increased and the number of cases began to decline, treatment had begun as usual with strict standard operating procedure (SOP) practices. The Ministry of Health, Malaysia has also guaranteed that medical treatments for non-COVID-19 patients will not be neglected to ensure that all patients receive the services they deserve. However, there are still some patients who refuse to come to the hospital for fear of being infected with COVID-19 on top of their current hepatitis C illness. Also, several patients preferred to stay at home and delayed or defaulted their treatment to prevent themselves to meet people on their way to the hospital (33). Therefore, the findings in this study reflected the scenario before the COVID-19 pandemic. Anecdotal information reported that this pandemic has consumed significant amount of the annual hospital budget for the management of COVID-19 itself, which resulted in the shrinking of other non-emergency services to compensate for the surging burden of COVID-19. Therefore, the estimated annual budget for hepatitis C from 2020 to 2022 in this BIA might be overestimated compared to the actual scenario in Malaysia.

At the moment, the analyses carried out and the results obtained are quite old. Due to the COVID-19 pandemic, the hepatitis C treatment scenario in Malaysia was halted and the estimated number of patients treated from 2021 to 2022 was significantly lower. Several factors were found to interfere with the treatment programme during the pandemic, including barriers to accessing treatment for people living with HCV due to the limited public health budget for treatment, which was exacerbated by the pandemic, limited staffing and infrastructure to initiate treatment, and disruptions in medical supplies and clinical care (33). This circumstance has further hampered the initiation of DAA treatment from 2021 to 2022. Therefore, the estimated number of patients in the last 2 years of the analysis may not reflect the actual situation in those years and the estimate may be extended for 2023 and 2024, while several containment and mitigation measures have been taken in Malaysia to flatten the peak of COVID-19 and ensure that the disease does not cause a huge burden on the health system. Several developed countries estimated that there was a significant decrease, ranging from 30 to 50%, in the initiation of treatment and testing for hepatitis C (34). While Malaysia, a developing country, is still far from achieving the global HCV elimination target despite the various treatment strategies, the decline could be more than 50%, hence the estimate for 2021 and 2022 can be used for the following 2 years in Malaysia.

While this study has assessed the impact of HCV infection on the healthcare cost from the provider perspective in Malaysia, it has several limitations that warrant caution in generalizing the findings. First, all costs in this study were based on healthcare resource utilization from the standard clinical pathway of hepatitis C management in Malaysia and the clinical framework for monitoring patients with DAA therapy from the WHO guidelines (21, 35). As a result, variations in clinical management due to clinical factors such as the presence of comorbidities and co-infection were not captured in this costing study. Nevertheless, the generalizability of the study findings might not be possible because of differences in healthcare systems and clinical characteristics of HCV patients from those of other countries. However, the methodology that has been used in this analysis was comprehensive and could be replicated by other researchers from other countries to estimate the financial implication of hepatitis C management.

Second, this study aimed to estimate the cost related to DAA therapy and disease stage monitoring only. The cost of HCV screening and diagnosis was not included. Currently, the majority of patients in care for HCV treatment in Malaysia have already been diagnosed and warehoused for DAA therapy (10). Even though the cost of HCV screening and diagnosis is beyond the scope of this study, the research team have conducted extended analyses to estimate the economic impact of both screening and diagnosis and the findings have been published elsewhere (31).

Thirdly, this cost estimate was based on one course of DAA therapy only. The efficacy of DAA therapy in achieving cure rates of nearly 100% suggests that treatment failure with DAA is very rare, and failures are commonly associated with poor treatment adherence and drug-drug interactions (21). In addition, all costs related to adverse events were excluded. Even though the clinical wellbeing of particular patients intra-treatment may necessitate frequent and more extensive utilization than is suggested in the clinical pathways, particularly with RBV, treatment with DAA therapy generally has very low rates of adverse events (21).

Finally, through the use of compulsory licensing, the government of Malaysia aimed to treat identified hepatitis C patients in stages based on the existing resources in healthcare facilities in Malaysia. This plan, however, will limit the number of treatment recipients to fewer than 20,000 over 5 years (11). Previously, hepatitis C patients in Malaysia were treated with interferon-based treatment before 2016. The planned entry for DAA into clinical practice led to many patients being warehoused in 2016 and 2017, with watchful waiting alongside regular clinical monitoring becoming common practice while awaiting the availability of DAA. In this period, some patients were recruited into clinical trials, while there were also patients who purchased the DAA drugs by their financial means. Due to the financial constraints and the constraints within the healthcare infrastructure, it is very challenging for Malaysia to meet the WHO elimination targets by 2030. Huge financial and resource investments are required to meet the HCV elimination targets. Findings from this analysis on the economic implications of chronic HCV disease can inform and facilitate resource allocation and healthcare decision-making to inform the development of a national investment case and the development of a sustainable hepatitis C care program in Malaysia.

In summary, the availability of affordable generic DAA drugs has enabled Malaysia to enhance access for HCV treatment nationwide. However, strengthening and consolidating a viable national HCV plan is important to address gaps in the cascade of care so that feasible policy strategies for the whole continuum of care nationally can be developed. This highlights the fact that accessibility of treatment is not the only essential component of healthcare services in this context; other services along the continuum of care also need to be strengthened in order to identify, refer and initiate treatment in a timely manner. While there is a need for policymakers to expand access to DAA treatment to the entire hepatitis C infected population, there is also a need to improve hepatitis C screening, and encourage early diagnosis and entry into care as part of the strategy to achieve the WHO targets (36).

The government has shown a strong political will to eliminate hepatitis C as a public health threat by the year 2030 through the use of DAA drugs and large-scale screening programs. Good collaborations between government and various advocacy groups provides opportunities to expand and scale-up screening, diagnosis and treatment of HCV in Malaysia through decentralizing HCV testing to primary health care centers and improving patient linkage to care (27). In conclusion, Malaysia needs to maximize and fully benefit the use of available generic HCV drugs and at the same time has to constantly improve the continuum of care to achieve HCV elimination as a public health therat by 2030.

## Data availability statement

The raw data supporting the conclusions of this article will be made available by the authors, without undue reservation.

## Author contributions

FS, AA, RM, and MD developed the concepts and researched and wrote the paper. FS, MD, AA, HJ, and SM performed the analyses. All authors had approved the final manuscript for submission.

## References

[1] daCosta DiBonaventura M Yuan Y Wagner JS L'Italien GJ Lescrauwaet B Langley P. The burden of viral hepatitis C in Europe: A propensity analysis of patient outcomes. Eur J Gastroenterol Hepatol. (2012) 24:869–77. 10.1097/MEG.0b013e3283551dee 22617367

[2] Patruni B Nolte E. A Projection of the Healthcare and Economic Burden in the UK. RAND Corporation report Hepatitis C. Prepared for the Hepatitis C Trust (2013). 28083286 10.7249/rhq-03-01-06 PMC4945231

[3] Gray E O'Leary A Kieran JA Fogarty E Dowling T Norris S. Direct costs of interferon-based and interferon-free direct-acting antiviral regimens for the treatment of chronic hepatitis C infection. J Viral Hepat. (2016) 23:677–86. 10.1111/jvh.12532 26996144

[4] Lee AS van Driel ML Crawford DHG. The cost of successful antiviral therapy in hepatitis C patients: A comparison of IFN-free vs. IFN-based regimens at an individual patient level in Australia. ClinicoEcon Outcomes Res. (2017) 9:595–607. 10.2147/CEOR.S146280 29042803 PMC5633296

[5] World Health Organisation. Global Hepatitis Report 2017. (2017).

[6] Iyengar S Tay-Teo K Vogler S Beyer P Wiktor S Joncheere K. Prices, costs, and affordability of new medicines for hepatitis C in 30 countries: An economic analysis. PLoS Med. (2016) 13:1–22. 10.1371/journal.pmed.1002032 27243629 PMC4886962

[7] Ho SH Ng KP Kaur H Goh KL. Genotype 3 is the predominant hepatitis C genotype in a multi-ethnic Asian population in Malaysia. Hepatobil Pancreat Dis Int. (2015) 14:281–6. 10.1016/S1499-3872(15)60363-0 26063029

[8] Mohamed NA Rashid ZZ Wong KK Abdullah SA Rahman MM. Hepatitis C genotype and associated risks factors of patients at University Kebangsaan Malaysia Medical Centre. Pak J Med Sci. (2013) 29:1142–6. 10.12669/pjms.295.3610 24353708 PMC3858944

[9] World Health Organisation. Progress Report on Access To Hepatitis C Treatment Focus on Overcoming Barriers in Low-and Middle-Income. (2018).

[10] Ministry of Health Malaysia. Press Statement Minister of Health 20th September 2017 – Implementation of the Rights of Government for Sofosbuvir Tablet to Increase Access for Hepatitis C Treatment in Malaysia. (2017).

[11] McDonald SA Azzeri A Shabaruddin FH Dahlui M Tan SS Kamarulzaman A. Projections of the healthcare costs and disease burden due to hepatitis C infection under different treatment policies in Malaysia, 2018–2040. Appl Health Econ Health Policy. (2018) 16:845–57. 10.1007/s40258-018-0425-3 30145775

[12] Cooke GS Andrieux-Meyer I Applegate TL Atun R Burry JR Cheinquer H. Accelerating the elimination of viral hepatitis: A lancet gastroenterology & hepatology commission. Lancet Gastroenterol Hepatol. (2019) 4:135–84. 10.1016/S2468-1253(18)30270-X 30647010

[13] Mohamed R Shabaruddin FH Azzeri A McDonald SA Dahlui M. Letter to editor: Hepatitis C elimination by 2030 in Malaysia: An achievable goal? J Viral Erad. (2019) 29:7. 10.1016/S2055-6640(20)30029-7 PMC6844403 31754450

[14] Mauskopf J Earnshaw SR Brogan A Wolowacz S Brodtkorb TH. Budget-Impact Analysis of Health Care Interventions. Springer (2017).

[15] Bilinski A Neumann P Cohen J Thorat T McDaniel K Salomon JA. When cost-effective interventions are unaffordable: Integrating cost-effectiveness and budget impact in priority setting for global health programs. PLoS Med. (2017) 14:1–10. 10.1371/journal.pmed.1002397 28968399 PMC5624570

[16] Sullivan SD Mauskopf JA Augustovski F Caro JJ Lee KM Minchin M. Budget impact analysis - Principles of good practice: Report of the ISPOR 2012 budget impact analysis good practice II task force. Value Health. (2014) 17:5–14. 10.1016/j.jval.2013.08.2291 24438712

[17] McDonald SA Mohamed R Dahlui M Naning H Kamarulzaman A. Bridging the data gaps in the epidemiology of hepatitis C virus infection in Malaysia using multi-parameter evidence synthesis. BMC Infect Dis. (2014) 14:564. 10.1186/s12879-014-0564-6 25377240 PMC4229598

[18] McDonald SA Dahlui M Mohamed R Naning H Shabaruddin FH Kamarulzaman A. Projections of the current and future disease burden of hepatitis C virus infection in Malaysia. PLoS ONE. (2015) 10:1–15. 10.1371/journal.pone.0128091 26042425 PMC4456147

[19] Azzeri A Shabaruddin FH Tan SS Dahlui M McDonald SA Kamarulzaman A. Clinical characteristics of patients with chronic hepatitis C infection at initial presentation to tertiary care in an asian middle-income country. Southeast Asian J Trop Med Public Health. (2018) 49:789–98.

[20] MAHTAS. Clinical Practice Guidelines Management of Chronic Hepatitis C in Adults. (2019).

[21] World Health Organisation. Guidelines for the Care and Treatment of Persons Diagnosed with Chronic Hepatitis C Virus Infection. (2018). 30307724

[22] Department of Statistics and Malaysia. Official Portal. (2015).

[23] Sui B Chang E Zhao K Qiu Y Hou W Chan P. Cost estimate and budget impact analysis of implementing the new guidelines for management of hepatitis C virus in China: A micro-costing study. Lancet. (2017) 390:S102. 10.1016/S0140-6736(17)33240-3

[24] Andrieux-Meyer I Cohn J Araujo de Araujo ES Hamid SS. Disparity in market prices for hepatitis C virus direct-acting drugs. Lancet Global Health. (2015) 3:e676–7. 10.1016/S2214-109X(15)00156-4 26475012

[25] Tanaka S Shinkawa H Tamori A Takemura S Uchida-Kobayashi S Amano R. Postoperative direct-acting antiviral treatment after liver resection in patients with hepatitis C virus-related hepatocellular carcinoma. Hepatol Res. (2021) 2021:13709. 10.1111/hepr.13709 34476874

[26] Drugs for Neglected Diseases Initiative. Efforts to Register New Hepatitis C Treatment in Malaysia Receive Support from Pharmaniaga. (2019).

[27] Drugs for Neglected Diseases Initiative. Drugs for Neglected Diseases Initiative and Pharco Pharmaceuticals to Test Affordable Hepatitis C Regimen with Support of Malaysian and Thai Governments. (2016).

[28] World Health Organisation. WHO Model List of Essential Medicines. (2017).

[29] Andrieux-Meyer I Tan SS Salvadori N Simon F Cressey TR Said R. Safety and efficacy of ravidasvir plus sofosbuvir for 12 weeks in non-cirrhotic and 24 weeks in cirrhotic patients with hepatitis C virus genotypes 1, 2, 3 and 6: The STORM-C-1 phase II/III trial stage 1 results. Int Liver Congr. (2018) 2018:8. 10.1016/S0168-8278(18)30459-8

[30] Suthar AB Harries AD. A public health approach to hepatitis C control in low- and middle-income countries. PLoS Med. (2015) 12:1–12. 10.1371/journal.pmed.1001795 25757228 PMC4355406

[31] Hiebert L Hecht R Soe-Lin S Mohamed R Shabaruddin FH Mansor SMS. A step-wise approach to a national hepatitis C screening strategy in malaysia to meet the WHO 2030 targets: Proposed strategy, coverage, and costs. Value Health Regional. (2018) 18:112–20. 10.1016/j.vhri.2018.12.005 30921591

[32] Khor V Poraviappan AA Azli SMZ Asri KMG Fahmy O. Experience from Malaysia during the COVID-19 movement control order. Urology. (2020) 4:7. 10.1016/j.urology.2020.04.070 32339556 PMC7195371

[33] Chan H-K Hassali MA Mohammed NS Azlan A Hassan MRA. Barriers to scaling up hepatitis C treatment in Malaysia: A qualitative study with key stakeholders. BMC Public Health. (2022) 22:1–9. 10.1186/s12889-022-12786-w 35189876 PMC8860373

[34] Kondili LA Buti M Riveiro-Barciela M Maticic M Negro F Berg T. Impact of the COVID-19 pandemic on hepatitis B and C elimination: An EASL survey. JHEP Rep. (2022) 4:100531. 10.1016/j.jhepr.2022.100531 35967191 PMC9364666

[35] World Health Organisation. Global Report on Access to Hepatitis C Treatment. Focus on Overcoming Barriers. (2016).

[36] Razavi H Robbins S Zeuzem S Negro F Buti M Duberg A-S. Hepatitis C virus prevalence and level of intervention required to achieve the WHO targets for elimination in the European Union by 2030: A modelling study. Lancet Gastroenterol Hepatol. (2017) 1253:1–12. 10.1016/S2468-1253(17)30045-6 28397696

